# Living Control Systems: Exploring a Teleonomic Account of Behavior in *Apis mellifera*

**DOI:** 10.3390/insects16080848

**Published:** 2025-08-16

**Authors:** Ian T. Jones, James W. Grice, Charles I. Abramson

**Affiliations:** 1 Applied Research and Analysis Company, Richmond, VA 23233, USA; ijones@aracscience.com; 2 Department of Psychology, Oklahoma State University, Stillwater, OK 74078, USA; charles.abramson@okstate.edu

**Keywords:** self-regulation, teleonomy, behavior, honey bees, insects, organism-centered, person-centered

## Abstract

Self-regulatory foraging behavior in honey bees (*Apis mellifera*) was investigated using the framework of Perceptual Control Theory (PCT). We developed a PCT-based model to describe how bees maintain goal-directed behavior, specifically targeting a sucrose-rich feeding site while overcoming a wind disturbance. In a controlled experiment, we found that 13 of 14 bees could successfully adjust their flight paths to overcome the disturbance and consistently reach the feeding target. While they demonstrated a great deal of individual variability regarding how they overcame the wind across experimental trials, they did so by finally adopting a headwind (i.e., flying into the wind) approach pattern rather than tailwind or crosswind approach patterns. These results support the application of PCT to the study of behavior in honey bees, which can be regarded as self-regulative (i.e., non-linear and dynamic) rather than as linear sequences of inputs and outputs. Given that such dynamic models are concerned with the functions or purposes of behavior, they may also be classified as teleonomic.

## Simple Summary

We investigated how honey bees (*Apis mellifera*) adapt their behavior when navigating to a sugar-rich food source in the presence of wind that disrupts their flight path. To explain this behavior, we applied Perceptual Control Theory (PCT), which posits that organisms actively pursue goals rather than merely respond to external stimuli. In our experiment, 13 of 14 bees successfully adjusted their flight paths to reach the food source despite the wind. The bees tested various approach strategies but ultimately favored flying directly into the wind, rather than allowing it to push them from behind or the side. Consistent with PCT principles, our findings indicate that honey bee behavior is self-regulating and adaptable, resembling a dynamic adjustment process rather than a linear cause-and-effect sequence. As PCT emphasizes the purpose of behavior, it can also be described as goal-directed (teleonomic).

## 1. Introduction

### 1.1. Living Control Systems: Exploring a Teleonomic Account of Behavior in Apis mellifera

Self-regulatory systems play critical roles in the physiological processes of organisms. These systems work to maintain specific homeostatic set-points, such as body temperature or blood glucose levels, which are essential for an organism’s optimal functioning and survival [1]. Regulatory systems also play important roles in governing the behaviors of organisms as they dynamically modify their actions on the basis of internal states and environmental stimuli [2]. Consider a common housefly, for example, perched on a pedestal in the middle of a drum marked with evenly spaced vertical lines. When the drum is rotated at different speeds by the experimenter, the fly will continuously adjust its behavior by rotating its body in the same direction and at the same speed as the drum, essentially maintaining an internal set-point of zero discrepancy between its movement and that of the drum [3]. As another example, consider a rat pressing a lever to receive a food pellet as a reward. As the experimenter increases the number of presses required to earn the reward, the rat will increase its rate of lever pressing accordingly while still maintaining a set rate at which it receives the reward [3]. In both examples self-regulatory systems are at work because the observed behaviors of the fly and rat are the result of a dynamic interplay between internal states and changing environmental stimuli. In the current paper we show how such systems can be modeled using the tools of Perceptual Control Theory and empirically studied in honey bees (*Apis mellifera*). We will also argue that self-regulatory behavioral systems are teleonomic and cannot be accurately understood otherwise.

### 1.2. Perceptual Control Theory

Explaining self-regulatory behavior is a particularly challenging task because it requires consideration of both the internal state of the organism as well as the feedback it receives from the environment. Simple behaviorist models run the risk of ignoring the former, while simple cognitive models run the risk of ignoring the latter. By “simple models” we here mean linear causation, input–output models (e.g., S → O → R) that fail to capture the complex and dynamic nature of regulatory systems. Examples and critiques of such models can be found in [2,4,5,6]. In more sophisticated language simple models can be said to ignore the function of behavior, which is to say they ignore the teleonomic aspects of behavior. William (Bill) Powers sought to correct the shortcomings of these input–output models with the introduction of his Perceptual Control Theory in the 1960s [5,7,8,9]. Building on cybernetic theory (e.g., [10,11,12,13]), Powers developed an intricate approach to modeling behavior with negative feedback loops which could also be organized into hierarchies to explain complex behavior. He explicitly made room in his approach for both the environment and the internal state of an organism, understanding behavior as an organism controlling its perceptions while it interacts with and receives feedback from the environment. Powers also regarded behavior as fundamentally teleonomic: “Behavior can be seen as purposive or goal-directed if it is recognized that the purpose of any act is to resist disturbances and that the reference condition describes the goal of the behavior.” ([5], p. 54).

The essential features of a simple negative feedback loop can best be demonstrated via an artifact, namely, a thermostat. The input device on the modern thermostat senses and analyzes the current temperature in the environment and computes whether the current temperature of the environment matches the ‘internalized’ temperature set by the homeowner. In moments when the room temperature does not match the set temperature, an error signal is computed, and an electrical signal is sent by the thermostat to the heating/cooling unit which warms or cools the air. The thermostat continues monitoring the air and compares the room and set temperature values, turning off the heating/cooling unit when the two values match. From the perspective of Perceptual Control Theory, the thermostat is a negative feedback control system, the function—or teleonomy—of which is to regulate the temperature in the room.

**Figure 1:**
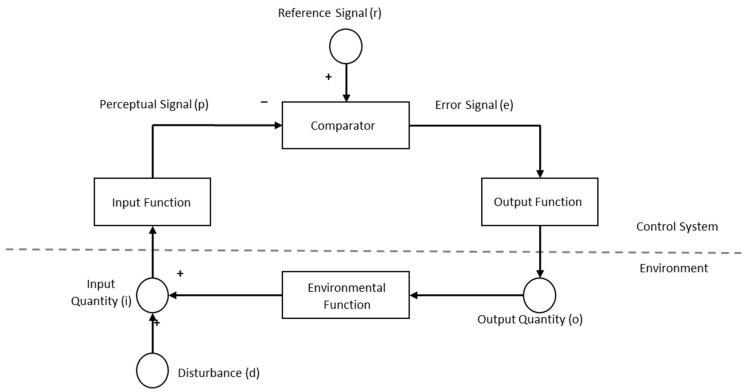
A Generic negative feedback loop for Perceptual Control Theory.

Figure 1 shows a model developed by Powers [5] as a generic and adaptable means for visualizing such control systems. Applying this model to the thermostat, the “Input Function” is the sensor on the thermostat that generates a value (p) sent to the “Comparator.” The “Reference Signal” (r) in the model is the temperature set by the homeowner, and the “Error Signal” (e) is the discrepancy between the room’s current temperature and the set value as determined by the comparator. The “Output Function” transforms the error signal which is sent to the heating/cooling unit as the “Output Quantity” (o) which determines its action (heat, cool, off). The action of the heating/cooling unit impacts the environment, the “Environmental Function,” and changes the temperature, the “Input Quantity” (i). The input quantity, then, is the moment-to-moment room temperature, and the “Disturbance” (d) is anything which can act upon the input quantity (e.g., outside weather, a hot oven, an open window). The negative feedback relationship continues so long as error is produced between the perceptual signal and referent signal. So long as the room temperature does not match the set temperature on the thermostat, the heating/cooling unit will act on the environment and counteract the disturbances.

What the control model shows is that the observed output to the environment (i.e., the cold or hot air blowing) is not a result of the input (i.e., elevated room temp) alone, but is rather the result of the discrepancy between the input signal (i.e., the current temperature) and the desired temperature (i.e., the pre-specified temperature); in other words, there is a negative feedback relationship between the inputs and the outputs. A linear causal model is simply not sufficient for explaining the behavior of the thermostat nor the observed changes in the variables measured in the system. Powers [5] believed that such negative feedback relationships can similarly be used to explain the simple and complex behaviors of organisms. Unlike machines, however, which have values programmed into them by external agents (e.g., a homeowner setting a thermostat), the organisms themselves maintain the prespecified values and defend those values from disturbances (i.e., things in the environment) which would otherwise cause a misalignment between the actual and ideal values. A given behavior is therefore not to be understood as a linear chain of inputs and outputs, but rather as an output produced by the organism to defend or counteract disturbances which produce misalignments (i.e., errors) to internalized variables (i.e., the controlled variables).

Even relatively simple behaviors such as the common knee jerk can be understood from the perspective of Perceptual Control Theory. As described by Marken [2], a teleonomic explanation of this behavior is that the quadricep muscle has a predetermined state of relaxation or stretch when at rest at a 90° angle. When the doctor strikes the tendon while the leg is in this position, the quadricep muscle’s state of stretch is disturbed. In other words, the hammer strike disturbs the degree of stretch in the quadricep muscle, and as a result the muscle resists it by contracting the quadricep and relaxing the hamstring. The resultant leg kick occurs because the muscle is resisting disturbance, or the stretching of the muscle produced by the tap (see [2,14], for more details). What appears to be a simple stimulus-response sequence of events is instead a control system at work.

In Perceptual Control Theory, the focus rests upon identifying the controlled variables, which are the variables the organism seeks to preserve at specified levels or amounts. The controlled variables have established reference signals, which are the pre-specified levels (i.e., set-points) at which they should be maintained. The controlled variables are identified when the organism behaves to counteract a disturbance, meaning that a particular controlled variable can only be identified by disturbing it. If the organism changes its behavior due to a disturbance, then the variable identified can be said to be ‘under control’ and labeled as a controlled variable.

Perceptual Control Theory has been successfully used to explain behaviors across a wide variety of species, including humans [9,15,16], rats [2,3,17,18,19,20], flies [3], German yellow jackets [21], crickets [18], jelly fish [22], and flat worms [23] as well as in contests between animals from the same species [24].

### 1.3. Current Experiment

In this study, we modified an operant conditioning paradigm to demonstrate how Perceptual Control Theory can be used to explain aspects of the foraging behavior of honey bees. Specifically, we trained bees to land on a rewarding target and then introduced a disturbance (viz., wind) to impede their approach to the target. The rewarding target was a 50% (by volume) sucrose solution placed on an orange dot. As a species, honey bees are foragers, meaning they must search for their food if they are to survive and reproduce. In accord with their nature, we trained the bees to repeatedly fly to, land upon, and feed from a rich food source.

From the perspective of Perceptual Control Theory, the distance between a bee and the food source is a control variable in which the ideal value is zero. In Figure 1 this is the Reference Signal (internal standard) of the bee. As the bee approaches its target, the Comparator returns a relatively large Error Signal, and various physiological mechanisms are consequently activated (Output Function) to produce observable flight (measured velocity, altitude, and direction; Output Quantities) toward the target (Environmental Function) as the measured distance (Input Quantity) is reduced. Our introduction of wind is a Disturbance the bee must overcome to reach the target. As shown in Figure 1, the Disturbance is part of the environment that affects the Input Function, the Perceptual Signal, and then the Comparator (completing the loop). To test for the presence of the control variable, we systematically altered the direction of the wind and recorded the behaviors of the honey bees. In accord with Perceptual Control Theory, we expected the bees to vary individually in the ways they overcame the disturbance, but to behave predictably and consistently in ultimately reaching the rewarding feeding target (i.e., the goal).

## 2. Materials and Methods

### 2.1. Subjects

**Figure 2:**
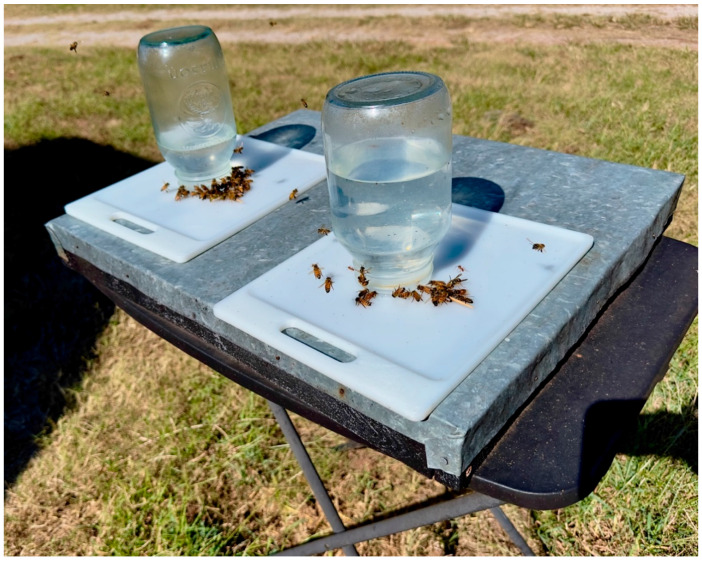
Free flying honey bee feeding station. Note. The feeding station was 13.41 m away from the hive and 5.49 m away from the training/experiment table. The mason jars contain an 8% (by volume) sucrose solution.

The subjects were 14 experimentally naive honey bees (*Apis mellifera ligustica*) from two maintained hives located in central Oklahoma. The two colonies were housed in a standard two-box Langstroth hive consisting of approximately 20,000–40,000 bees. The hives were located in a pesticide-free area, and no chemicals were applied to the colonies for mite control. Bees from these two colonies and perhaps other bees in the vicinity regularly foraged at the feeder which was placed 44 feet (13.41 m) from the colonies. The colonies were approximately two years old and were obtained from an apiary. During the experiment, one of the colonies absconded. However, we are under the impression that bees from the absconded colony were still foraging on the feeder, as the number of foraging bees did not appear to decrease. Individual foragers were grabbed from a feeding station (see Figure 2) in a small matchbox and brought to a separate table. Throughout this experiment the bees were treated in accord with the American Zoological Association’s ethical guidelines for research with animals. Oklahoma State University does not require an Institutional Animal Care and Use Committee (IACUC) review for research conducted with invertebrate animals.

### 2.2. Apparatus

**Figure 3:**
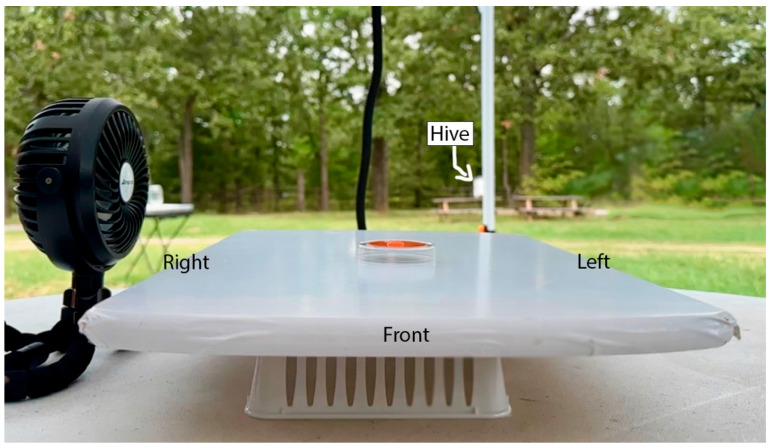
Experimental testing table setup. Note. The training/experiment table was a white circular table that is approximately 18.29 m away from the hive and 5.49 m away from the feeding station (see Figure 2). The placement of the fan is always in reference to the hive (featured). The orange target is shown in the Petri dish.

The landing site for the experiment was placed atop a white circular table approximately 18.29 m from the hive and 5.49 m from the feeding station. As can be seen in Figure 3, the landing site was a 45 cm × 31.5 cm × 1.8 cm wooden board placed on top of an upside-down, white plastic basket (dimensions: 25.8 cm × 16.3 cm × 5.9 cm). The wooden cutting board was covered in white contact paper (brand: EasyLiner Contact Paper) to maintain a neutrally colored landing site. The bees fed from a 6 cm (diameter) clear, circular Petri dish. For the initial shaping trials a 6 cm (diameter) laminated gray (brand: Glidden, Color and Code: Granite Grey, 00NN 37/000Glidden, Pittsburgh, PA, USA) target was affixed to the bottom of the Petri dish, and a 25 mL droplet of 50% sucrose solution was placed on the center of the gray target. For the three control trials and nine experimental trials the gray target was replaced with a 4.5 cm (in diameter) circular, orange (brand: Valspar color chip, Autumn Blaze, #2002-1A; hex: #e55b2b, Valspar Corporation, Cleveland, OH, USA) target upon which the sucrose solution was placed.

The landing site was positioned in reference to the hive, such that the back of the landing site was always facing the hive (see Figure 3). The target was placed 12.6 cm away from the left and right sides, and 19.5 cm away from the front (as measured from the center of the target to the edge of the landing site). In addition, one small, black, circular, stroller fan (brand: AMACOOL, Shenzhen LuanSheng Electronic Technology Co., Ltd, Shenzhen, China) was placed 20 cm away from the center of the target in one of three different positions (see below). The fan was approximately 12 cm in diameter and stood 13.5 cm tall, as measured from the center of the fan to the table. The fan had three levels of varying speeds, 2 m/s, 3.1 m/s, and 3.6 m/s, respectively. These speeds were measured 20 cm away from the center of the fan using the averaging function built into a digital anemometer (brand: BTMETER BT-100 Handheld Anemometer, BTMETER,) over a minute long interval. The third level, 3.6 m/s, was used in this experiment, and the speed of the wind was checked between each trial when the bee was not present to ensure that approximately 3.6 m/s was achieved for each trial.

### 2.3. Procedures

Following well established protocols (e.g., [25,26,27,28]), the honey bees were randomly pulled while feeding from the 8% feeder table and captured in matchboxes. Once captured, the bees were brought to the experimental table and slowly released from the matchbox while feeding from the 50% sucrose (by volume) solution that was placed on the neutral gray training target. While feeding, the bees were marked on their abdomen, thorax, or both with “OPI” brand nail polish. Four colors (big apple red: #NLN25, mi casa es blue casa: #NLM92, exotic birds do not tweet: #NLF91, that’s hula-rious: #NLH65) of OPI Nail Lacquer (OPI Products Inc., Calabasas, CA, USA) were used as up to four bees were simultaneously observed. Since many bees did not return to the table after their first visit during the two acquisition (i.e., shaping) trials, training multiple bees made data collection more efficient. Once the bee had been marked it was allowed to fly back to the hive and return to the neutral gray target two times before beginning the experimental phase of the study. During these two trials the fan was placed 20 cm to the right of (relative to the hive, see Figure 3) the gray training target but was not turned on. Once the bee completed the two shaping trials, the gray target was replaced by the orange target to begin the experimental phase which consisted of three control and nine experimental (12 total) trials. All trials were recorded with a GoPro Hero 10 camera (GoPro, Inc., San Mateo, CA, USA) set to 1080p, 60FPS, and a linear lens setting. A flex-arm camera mount held the camera in place, but no set position was used across all days of the experiment. Top (overhead) and side angles were used from which the three behavioral variables (see below) could easily be coded.

During these trials the fan was placed in three positions: at the front, to the left, or to the right of the orange target. During the three control trials the fan was kept off so that the bee could learn to interact with the orange target without the disturbance of the wind from the fan. In other words, the first three trials were similar to the shaping trials where the bee learned the orange target is the desired target upon which to land. The fan was turned on after these three trials were completed. The fan’s starting position varied by group and stayed in the same position for three consecutive trials before being moved to the next position. In total there were six groups to control for the positionality of the fan (see Table 1). After the first three trials and based on their assigned grouping, each individual bee experienced three trials with the fan at the front, three trials with the fan at the left, and three trials with the fan on the right side of the landing site. At all times the fan was positioned 20 cm away from the center of the landing target.

### 2.4. Behavioral Coding and Dependent Variables

There were three primary dependent variables in the current experiment: (1) landing successes, (2) number of approaches to the target, and (3) direction of approaches. The landing successes were recorded on site during the experiment whereas the approach observations were taken from video recordings of the trials. Video recordings were used to tally the number of approaches and how the bees approached the target. A successful landing was defined as the bee landing and feeding from the 25 mL drop of 50% sucrose solution. Accordingly, we expected one successful landing per trial, unless the bee landed, fed, and then lifted off and re-landed and fed because something interfered with the bee while it was feeding from the sucrose solution. It should be noted that the camera overheated on several occasions, resulting in the loss of data for several trials for four honey bees (cases #5, #8, #9, and #11).

The bees varied in the number of times and in the manner which they approached the target prior to landing and feeding. Upon reaching the table the bees would hover, approach the target, retreat, change position, and move toward the target again, sometimes from different directions. Observing this behavior on the videotapes, an approach was tallied when the bee’s head was positioned towards the target (i.e., directed at it) while simultaneously flying in the wind stream. Approach direction was recorded with four possible outcomes: (1) headwind, (2) tailwind, (3) left, (4) right. Headwind meant the bee was approaching the target into the wind. Tailwind meant the bee was approaching the target with the wind. Left and right meant that the bee was approaching from either the left or right side of the table relative to the hive (see Figure 3), respectively.

Lastly, the intertrial intervals were also recorded from the moment the bee left the table to the moment it landed and received the reward. A limit of 20 min was placed on each bee to return to the feeding table. Only one bee (case #11) failed to return within this time period after the 11th trial. Data for this bee are nonetheless included in the analyses below since it had completed 92% of the total trials. Upon completion of the 12th trial, each bee was captured and sacrificed to ensure it was not reused and that every bee was experimentally naïve.

## 3. Results

### 3.1. Successful Landings

Having trained to fly to the table and feed upon the sucrose solution, could the bees overcome the added wind disturbance and still reach the target? To answer this question, we analyzed the recorded number of landing successes for each individual honey bee. Specifically, we tallied the number of successes across the three control trials (1–3) and separately across the experimental trials (4–12) and converted the results to percentages; viz., the Percent Correct Classifications (PCC) indices. A randomization test was then conducted for each analysis to determine a plausibility value, referred to as a chance value (*c*-value; see [29,30]), which aids in assessing whether or not the observed PCC index can be attributed to physical chance. Like traditional *p*-values, low *c*-values (e.g., <0.05) are desirable to support a causal interpretation of the outcome.

Table 2 presents the results from these analyses, and as can be seen, the PCCs for 13 out of 14 bees were 100% (*c*-values < 0.001) for both the control and experimental trials, therefore indicating they successfully landed on the target and fed from the sucrose reward on every trial, with or without the presence of wind. Aggregating across all trials and all bees, the bees were successful on 159 out of 167 trials; PCC = 95.21%, *c* < 0.001. Only one bee (case #11, see Table 2) was unable to consistently land on the target once the wind was turned on. This bee was able to land and feed during the first three trials, but once the wind was turned on it never successfully landed on the target. This bee also failed to return to the table on the 12th trial and was discontinued after 20 min as defined in the methods.

### 3.2. Approaches and Direction

**Figure 4:**
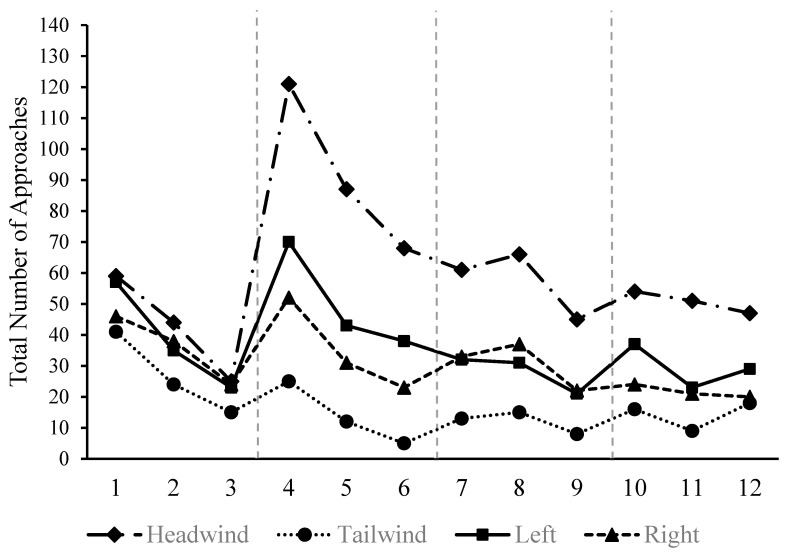
Total number of approaches across all bees and for four approach directions. Note. Dotted vertical lines demarcate changes in the fan’s position. During Trials 1–3 the fan was off. During trials 4–12 the fan was on but was rotated to three positions (right, front, or left) every three trials (i.e., Trials 4–6, 7–9, and 10–12).

Examination of the recorded videos revealed that the bees varied the rates (i.e., their speeds) at which they flew through the wind as well as the direction from which they approached the target. The bees could also be seen pushing against the wind, being pushed back, and then pushing forward again through the wind. These behaviors can readily be seen in a number of example videos posted in an online repository (Video Example X (https://drive.google.com/file/d/1zb-OJtuMbTlyamHSAtK6GlngumXqIwLa/view?pli=1, uploaded on 9 July 2025; last accessed on 15 August 2025) & Video Example Y (https://drive.google.com/file/d/1Fn6he857-UY2DHK6f-wK84G-xnsCmKQg/view, uploaded on 9 July 2025; last accessed on 15 August 2025). Given this variability, were the bees observed to demonstrate a general preference in the direction they approached the target? For descriptive purposes only, we first tallied the number of approaches across all bees for each of the four directions: into the wind (i.e., headwind), with the wind (i.e., tailwind), from the left side of the target (i.e., left), or from the right side of the target (i.e., right). As can be seen in Figure 4, across the first three trials in which the fan was turned off, the bees tended to reduce the number of approaches needed to land on the target. Importantly, while the tailwind approach was used less frequently than the others, no strong preference is visible in the plotted totals. When the fan was turned on for the remaining experimental trials, however, a clear preference for the headwind approach was observed across the nine experimental trials, while the tailwind was generally avoided. Similarly to when the fan was turned off, the bees also tended to require fewer approaches to successfully land on the target from the fourth to the twelfth trial. For the first experimental trial (Trial #4; see Figure 4), all fourteen honey bees used more headwind approaches than tailwind or right side approaches (PCCs = 100%, *c*’s < 0.01). Eleven of the bees used more headwind approaches than left side approaches (PCC = 78.57%, *c* = 0.01). By the final trial (Trial #12), the majority of bees still used the headwind approach more frequently than the tailwind (PCC = 69.23, *c* = 0.03), right side (PCC = 76.92, *c* = 0.01), or left side (PCC = 76.92, *c* < 0.01), although the results were not as pronounced.

As a general test of the headwind preference across all twelve experimental trials shown in the aggregate tallies in Figure 4, we constructed a logic statement for the number of approaches. The logic statement was classified as ‘true’ if and only if the number of headwind approaches was greater than the number of tailwind approaches, the number of right side approaches, and the number of left side approaches for a given trial:

True ↔ (H_k_ > T_k_) ∧ (H_k_ > L_k_) ∧ (H_k_ > R_k_),

where ∧ represents logical conjunction, _k_ represents the paired *k*th trial, and H, T, L, and R represent ‘headwind,’ ‘tailwind,’ ‘left,’ and ‘right,’ respectively. For example, the third bee (case #3) approached the target 16 times on trial #4: eight times into the headwind, two times with the tailwind, and three times each from the left or right directions. The logical statement, (8 > 2) ∧ (8 > 3) ∧ (8 > 3), was therefore true for this particular bee and trial. Its number of approaches into the headwind was greater than its number of approaches for each of the other three directions. The number of times the logical statement was true across the three control or the nine experimental trials was tallied for each bee and reported as Percent Correct Classifications indices and evaluated with randomization tests.

Table 3 presents the results for the first three control trials (1–3) and the nine experimental trials (4–12). For the first three trials, since the fan was not on, the bees were not expected to show a clear directional preference. Results supported this expectation as only three bees (cases #1, #6, and #7) showed a slight preference (PCCs = 66.67%) for approaching the target while facing the fan. Aggregating across all of the bees, only 9 out of 41 trials were classified as ‘true,’ yielding a PCC equal to 21.95%, *c* = 1.00. During the first 3 trials, then, the bees did not prefer to fly towards the fan (which would be the headwind if the fan was on) more than the other approaches when landing on the target.

For trials 4 through 12, however, the bees’ preferences changed. As can be seen in Table 3, six bees showed strong preferences for the headwind approach (PCCs ≥ 75%, c’s ≤ 0.15), while another six bees showed moderate preferences for this approach (PCCs > 62%, c’s ≤ 0.26). Only two bees (case #5 and case #11) failed to show a clear headwind preference (i.e., PCC ≤ 50%) in their approach preferences. Across all bees 84 out of 121 observations matched expectation (i.e., were true for the logic statement), yielding a PCC equal to 69.42%, *c* < 0.0001. These results reveal that when the wind was turned on, the bees approached the target into the wind (i.e., headwind) more than any other direction. It should also be noted that *c*-values, like traditional *p*-values, are influenced by sample size and the number of possible outcomes (in this case two: true vs. false). With a larger number of trials or more possible outcomes, the *c*-values for the individual PCCs magnitudes above would be smaller.

Finally, we evaluated the direction of the last approach each bee made before it successfully landed on and fed from the target. We specifically tallied the number of times the successful approach was from the headwind direction rather than from one of the other three directions. Results revealed PCCs equal to 100% (*c*-values < 0.0001) for ten of the fourteen bees; meaning, these bees successfully landed on the target by approaching into the wind (i.e., a headwind) across all of the trials that the wind was turned on (i.e., trials 4–12). Recall from above that one bee (case #11) made many attempts to land, but never successfully landed. Aggregating across all bees and trials when the fan was on, the bees landed successfully by using a headwind approach on 111 out of 114 trials; PCC = 97.37, *c* < 0.0001. There were only three instances across all trials and all bees during which the bees successfully landed using a direction other than the headwind approach. Cases #2 and # 14 each made one successful landing after approaching from the left and case #7 made a successful landing after approaching with the wind (i.e., tailwind), though video analysis revealed this to be a rather haphazard landing.

## 4. Discussion

Self-regulatory systems are best understood as structures and processes organized into non-linear, dynamic wholes. Well known examples include the positive feedback loop mechanism underlying blood clotting and the negative feedback loop mechanism underlying body temperature regulation [31,32,33]. Behavior, too, can be modeled in ways that go beyond the traditional linear causation, input–output approach. Drawing upon the tenets of Perceptual Control Theory we designed our study so that honey bees would have to dynamically modify their behavior to counteract a disturbance and reach a rewarding target. More specifically, the bees had to modify the speed of their flight and the direction of their flight paths to counteract wind disturbances from varying directions in order to land on a target and receive a sucrose reward. If behavior is regarded as the control of perception, then identifying and manipulating a controlled variable is an important part of experimentation (see also [2]). In this case the controlled variable was the distance between the bee’s current position and the target, and the ideal distance was a value of ‘0’ (i.e., no distance from the target). In accord with the negative feedback loop in Figure 1, the bees achieved this internal standard by varying the direction which they approached the target and the speed of their flight to overcome the 3.6 m/s wind and reach the target.

Results revealed the bees preferred to approach the target by flying into the wind as a means for counteracting the disturbance. The fan position was moved (i.e., disturbed) every three trials to a new position, but regardless of the position the bees still approached into the wind more than any other direction; meaning, the bees were not approaching the target consistently from the same direction (e.g., from the back side of the landing platform); but rather, they varied their approaches to match a preferred approach (viz., approach into wind). From the observational data, the bees were seen varying the speeds with which they were flying, pushing against the wind to get closer to the target, getting pushed back, and then pushing forward again and eventually landing. Across almost all the trials (97.37%), the bees approached the target into the wind (i.e., headwind) on their final approach before successfully landing and feeding on the target. It is important to point out that no two bees showed identical strategies in how they eventually approached the target facing into the headwind. Some bees tried various approaches from different directions and altitudes while others quickly oriented themselves into the headwind. In other words, while the bees varied with regard to their individual flight paths and speeds, nearly all overcame the disturbance and eventually reached the target through the headwind. The one honey bee (case #11) that never landed successfully on any of the experimental trials showed no preference for a headwind approach.

Individual variability in the specific actions of organisms as they strive toward their goals is common in studies of complex behaviors, such as foraging. Perceptual Control Theory makes room for this variability in a number of ways which can be explained by returning to and considering Figure 1 further. The model in the figure is necessarily simplified and is sufficient for explaining the patterns of results in this study. A more complete model, however, would include multiple levels of hierarchically controlled variables (see [2,5,6,9]). During flight the bees must also control their stability by varying their body position (i.e., pitch and yaw). Consistent with the fundamental laws of aerodynamics, by approaching into the wind the bees will have greater stability when landing, the same as an airplane approaching a runway. Similarly, it is easier to respond to and counteract harsh changes (e.g., a crosswind) when flying into the wind than it is in other directions. Finally, flying into the wind (whether landing or taking off) has a well-known impact on lift and drag, which are important variables for flight (see [34,35,36]). In any given moment, each bee is simultaneously controlling all of these variables (among others) in harmony and in the context of continuously changing environmental feedback. With such complexity, individual variability is to be expected.

Generally speaking, results from studies regarding the flying dynamics of honey bees comport well with PCT. For instance, when flying through tunnels, bees maintain equidistance from the walls by balancing perceived image speeds, controlling flight position to match a centered reference [37]. Flight speed is regulated by holding average image velocity constant, reducing collision risks by stabilizing optic flow perception. Most relevant to the present work is the finding that honey bees adjust their speed to land at a slow speed, thus avoiding a crash. During landing, bees attempt to keep the rate of expansion of the visual image constant, thus slowing down to near-zero velocity at touchdown [38] and showing another form of the control of perceptual inputs, consistent with PCT. Distance to food sources is gauged by integrating optic flow, maintaining a visually driven ‘odometer’ that is robust to wind and energy variations [39]. These behaviors rely on distinct movement-sensitive mechanisms, separate from the optomotor response, suggesting multiple control systems managing the perceptual variables for navigation (see [36] for a review of these self-regulation behaviors). In different terminology, these navigational feats can be construed as the operation of servomechanisms [40] which are entirely consistent with Powers’ PCT.

Figure 1 also omits the fact that multiple disturbances are most certainly at play for any given bee and trial. We controlled the wind disturbance with a fan, but other disturbances impacted each bee as it left the hive and flew towards the target. For example, the bee may have needed to navigate around other bees to successfully leave the hive, which would change its flight path to some degree. Other bees may also have been in the flight path, particularly when leaving the hive. While the experiment was conducted on days without strong winds, the wind likely fluctuated between trials and even when the bee was in flight. The appearance of the table, landing site, and reward likely fluctuated as well from cloud cover and the movement of the sun. All of these potential disturbances had to be counteracted by the bee as it left the hive and flew to the table. Again, Perceptual Control Theory makes room for these disturbances which are not under the control of the experimenter [19]. If the organism is controlling its perceptions, it will reliably reach its goal although not exactly the same way. As William James pointed out over one hundred years ago, “fixed end…varying means!” (as quoted in [6], p. 86).

More generally, Perceptual Control Theory makes room for greater nuance in modeling behavior because it fits under the umbrella of Aristotelian philosophy. Iconic models, like the image in Figure 1, have played important roles in the history of scientific discovery [41,42,43]. One need only think of ball-and-stick models of molecular compounds or Watson and Crick’s famous double-helix model of Deoxyribonucleic Acid. Such models are explanatory in nature as they encapsulate the causal structures of the natural systems being studied. For Aristotle, there are four fundamental causes, or explanatory factors, that may be incorporated into a scientific explanation. Material cause refers to the physical substance or matter of which something is made, for example, the marble of a statue. Formal cause refers to the design, structure, or nature that defines what a thing is, such as the shape and features that make a statue recognizable as that of a man. Efficient cause identifies the agent or process that brings something into being or changes it in some important way, such as the numerous strikes of the sculptor’s hammer. Finally, final cause is the purpose or end goal for which something or some process exists, such as the sculptor’s reason for creating the statue or the function of foraging in honey bee behavior. Nuance can be added to discussions of final cause by using the word “teleology” to describe human goals and purposes, and the word “teleonomy” to describe the functions and end states found in the physiology and behavior of organisms as well as in other natural systems [44,45,46,47]. In sum, the four causes–matter, form, agency, and purpose–offer a comprehensive and holistic way of explaining systems in nature.

Perceptual Control Theory is teleological or teleonomic because it explicitly incorporates final causes (purposes, goals, internal standards, end-states) into its modeling approach (see [2,3,48]). Every control system will incorporate at least one internal set-point which serves as its goal or natural end state. From the perspective of the observer, this set-point is central to understanding or explaining the system. Any mention of the function of the system, for instance, will entail its final cause (e.g., the function of the immune system is to ward off and fight infection). The function of the negative feedback loop considered in this study is to guide the bees through disturbances so they may reach a food source–the goal or end point. Guided flight to a specific target is part of a larger system, foraging behavior, the purpose of which is the well-being of the individual bee as well as the success and survival of its colony and species. In a way, the negative feedback loops incorporated in Perceptual Control Theory are organized around their final causes. It is as if the final cause holds the system together, and by systematically varying disturbances, as was performed in the current study, such causes can be identified and studied.

The word “system” implies not just final causes, however, but structures and processes organized into a coherent whole—a Gestalt. Honey bees are of a particular nature (formal cause) which entails foraging behavior. Not all organisms, of course, are foragers, and hence the current study relied upon the given nature of honey bees for its design. As noted above, pointing out what might seem obvious nonetheless enhances our explanation of the bees’ successful navigation through the disturbances to reach the target. Material causes can also be invoked in discussions of the sucrose solution, a bee’s proboscis, eye structure, neuroanatomy, etc., to deepen our understanding of its behavior. Lastly, efficient causes are part-and-parcel of negative feedback loops as can be seen in the arrows in Figure 1. The arrows represent time-ordered sequences of changes in the system; for example, the Input Function sends a signal to the Comparator which then sends a signal to the Output Function. In this way, we can see that simple input–output models (e.g., S → O → R) can be subsumed by more holistic, dynamic models rooted in Aristotle’s philosophy of nature.

Approaching the study of behavior from this vantage point may lead to new ideas and to new ways of understanding existing ideas or theories. For example, ‘effort’ and ‘motivation’ have always been of interest to the neo-behaviorists (e.g., [49,50,51]). In developing and considering previous experiments from the vantage point of Perceptual Control Theory, a novel methodology to test ‘effort’ in honey bees could be employed. The fan in the current study could be systematically varied to find the point at which the organism will stop attempting to land on the target. The fan speed was kept at 3.1 m/s, but the speed could be intensified on each trial to determine the point at which a bee would no longer attempt to land and leave, so long as the maximum speed does not exceed its maximum flight speed capabilities. Such research would also benefit from more precise measurements of flight speeds and approach vectors by filming the bees from a set top position. With a circular area mapped onto the landing site, exact measurements could then be taken and additional navigational quantities computed (see [52] for a review of research focused on insect navigation). The reward could also be varied to determine if the bees are responding to a specific ‘quality’ of reward (see [53,54]). More generally, systematic variation and rigorous control procedures are methods which were developed and are routinely employed by comparative psychologists. Accordingly, Perceptual Control Theory may breathe new life into a field that is wrongly considered by many to be outdated [55].

In summary, scientists need not think that behaviors are a byproduct of blind, trial and error processes (see [5]); rather, they may be understood as teleonomic…as reaching ends with regularity. If Yin [3] is correct in suggesting that a “…restoration of purpose is therefore a new beginning for the sciences of behavior (p. 345),” then Perceptual Control Theory and other tools for modeling the dynamics of complex organic systems may lead to new ways of studying honey bee behavior. With an increase in our understanding of honey bee behavior, we scientists can develop more effective strategies to protect pollinators, enhance agricultural productivity, and mitigate threats like colony collapse disorder and environmental stressors.

## Figures and Tables

**Table 1:** Fan positions.

Trial	Group					
	1	2	3	4	5	6
1	F	F	L	L	R	R
2	F	F	L	L	R	R
3	F	F	L	L	R	R
4	F	F	L	L	R	R
5	F	F	L	L	R	R
6	F	F	L	L	R	R
7	L	R	F	R	F	L
8	L	R	F	R	F	L
9	L	R	F	R	F	L
10	R	L	R	F	L	F
11	R	L	R	F	L	F
12	R	L	R	F	L	F

Note. During the first three trials (above the solid line) the fan was turned off and was placed in the same position as when the fan was turned on for trial four. The fan was placed in one of three positions (R = Right, F = Front, L = Left), as shown in Figure 3.

**Table 2:** Successful landing results.

Case #	Trials 1–3			Trials 4–12		
	Trials	PCC	*c*-Value	Trials	PCC	*c*-Value
1	3	100.00	0.123	9	100.00	0.002
2	3	100.00	0.126	9	100.00	0.002
3	3	100.00	0.124	9	100.00	0.002
4	3	100.00	0.124	9	100.00	0.002
5	3	100.00	0.125	9	100.00	0.003
6	3	100.00	0.126	9	100.00	0.001
7	3	100.00	0.125	9	100.00	0.002
8	3	100.00	0.124	9	100.00	0.001
9	3	100.00	0.124	9	100.00	0.002
10	3	100.00	0.125	9	100.00	0.002
11	3	100.00	0.126	8	0.00	1
12	3	100.00	0.125	9	100.00	0.002
13	3	100.00	0.126	9	100.00	0.002
14	3	100.00	0.125	9	100.00	0.001
Total	42	100.00	<0.001	125	93.60	<0.0001

Note. The PCC is the percentage of trials the bee was able to successfully land and feed from the sucrose reward. The *c*-value is a proportion from a randomization test, and low values indicate the observed PCC index is not plausibly the result of physical chance.

**Table 3:** Headwind preference results.

Case #	Trials 1–3			Trials 4–12		
	Trials	PCC	*c*-Value	Trials	PCC	*c*-Value
1	3	66.67	0.51	9	77.78	0.09
2	3	0	1	9	66.67	0.26
3	3	0	1	9	88.89	0.02
4	2	0	1	9	66.67	0.26
5	3	33.33	0.87	8	50.00	0.64
6	3	66.67	0.50	9	77.78	0.09
7	3	66.67	0.51	9	66.67	0.25
8	3	0	1	8	75.00	0.15
9	3	0	1	8	62.50	0.37
10	3	0	1	9	66.67	0.25
11	3	33.33	0.87	7	42.86	0.77
12	3	0	1	9	66.67	0.25
13	3	0	1	9	77.78	0.09
14	3	33.33	0.88	9	77.78	0.09
Total	41	21.95	1.00	121	69.42	<0.001

Note. The PCCs indicate percentages of trials the bees showed a preference for the headwind directional approach over the other three options. Some bees have fewer trials because the video was unavailable to review the number of approaches. For trials 1–3 the fan was off; for trials 4–12 the fan was on.

## Data Availability

Data can be seen via the link: https://osf.io/4dt9p/ (accessed on 6 July 2025).
